# ﻿Three new species of the genus *Parandes* Muir, 1925 (Hemiptera, Fulgoromorpha, Cixiidae) from China, with an updated checklist and key to species

**DOI:** 10.3897/zookeys.1248.159068

**Published:** 2025-08-05

**Authors:** Sha-Sha Lv, Nian Gong, Xiao-Ya Wang, Lin Yang, Yu-Bo Zhang, Xiang-Sheng Chen

**Affiliations:** 1 Guizhou Key Laboratory of Agricultural Biosecurity, Guizhou University, Guiyang, Guizhou, 550025, China; 2 Institute of Entomology, Guizhou University, Guiyang, Guizhou, 550025, China; 3 The Provincial Special Key Laboratory for Development and Utilization of Insect Resources of Guizhou, Guizhou University, Guiyang, Guizhou, 550025, China; 4 Guizhou Provincial Engineering Research Center of Medical Resourceful Healthcare Products, Guiyang Healthcare Vocational University, Guiyang, Guizhou, 550081, China; 5 Anshun University, College Agriculture, Anshun, Guizhou, 561000, China

**Keywords:** Cixiids, distribution, identification key, Oriental region, planthopper, sap-sucking insects, taxonomy

## Abstract

Three new species of the genus *Parandes* Muir, 1925 (Hemiptera, Fulgoromorpha, Cixiidae, Andini), *P.elongatus* Lv & Chen, **sp. nov.**, *P.guangxiensis* Lv & Chen, **sp. nov.** and *P.hamatus* Lv & Chen, **sp. nov.**, are described and illustrated from Southwest China (Yunnan and Guangxi) to give the genus six species in total. An updated identification key and checklist to all known species of *Parandes* are provided, as well as a map of their geographic distributions.

## ﻿Introduction

Andini is a small tribe within the subfamily Cixiinae (Hemiptera, Fulgoromorpha, Cixiidae), which was established by Emeljanov (2002). Although it is formally classified within Cixiinae, recent molecular phylogenetic analyses place it within the cixiinian lineage, which likely also includes Brixiini. These findings suggest that the tribe constitutes part of a paraphyletic group that originated in the Lower Cretaceous (Bourgoin et al. 2023; Bucher et al. 2023; Luo et al. 2025).

The tribe comprises three genera (*Andes* Stål, 1866, *Andixius* Emeljanov & Hayashi, 2007, and *Parandes* Muir, 1925) and a total of 135 species (Bourgoin 2025). In China, 26 species belonging to this tribe have been recorded, including fifteen species in the genus *Andes*, nine species in the genus *Andixius*, and two species in the genus *Parandes* (Fennah 1956; Zhi et al. 2018; Wang et al. 2020, 2022, 2023a, b; Luo et al. 2022; Bourgoin 2025).

*Parandes* Muir, 1925 belongs to the tribe Andini, and is easily recognized from other members in this tribe by the head in profile with the junction of the vertex and frons slightly angular and slightly produced, and the fore coxa produced and rounded on the outer edge of the apical half (Muir 1925; Wang et al. 2023b). It is the smallest genus within the tribe Andini, comprising only three species, all of which are distributed in the Oriental region. Muir (1925) first described *P.simplus* Muir, 1925 from Indonesia (Löcker et al. 2007), which was designated as the type species. Then Wang et al. (2023b) described two new species from China, *P.circinatus* Wang & Chen, 2023 and *P.fuscus* Wang & Chen, 2023.

Herein, three new *Parandes* species, *P.elongatus* sp. nov., *P.guangxiensis* sp. nov. and *P.hamatus* sp. nov., are described from China. As a result, the number of species in the genus has increased to six, with five species recorded from China.

## ﻿Material and methods

The external morphology terminologies are as follows: male genitalia follow Bourgoin (1987), female genitalia follow Bourgoin (1993) and Chen and Zhi (2023), and wing venation follows Bourgoin et al. (2015). Dry specimens were used for the descriptions and illustrations. Body measurements are from the apex of the vertex to the tip of the forewing; vertex length (median length of vertex) was measured from the apical transverse carina to the tip of the basal emargination. Photographs of the adult habitus were obtained by the KEYENCE VHX-6000 and VHX-1000 systems, and multiple layers were stacked using Helicon Focus 6. External morphology and drawings were done under a Leica MZ 12.5 stereomicroscope. The photographs and illustrations were scanned with a CanoScan LiDE 200 and imported into Adobe Photoshop 6.0 for labeling and plate composition. The distribution map was generated with ArcGIS 10.7.

The type specimens examined are deposited in the Institute of Entomology, Guizhou University, Guiyang, Guizhou Province, China (**IEGU**).

## ﻿Taxonomy

### ﻿ Parandes

Taxon classificationAnimaliaHemipteraCixiidae

Muir, 1925

7B4FFBFD-791D-5ED5-9BE1-9C13E156E244

Parandes Muir, 1925: 511; Wang et al. 2023b: 112.

#### Type species.

*Parandessimplus* Muir, 1925, original designation.

#### Diagnosis.

For the diagnosis of *Parandes* see Wang et al. (2023b: 112).

#### Distribution.

**Figure 1.**
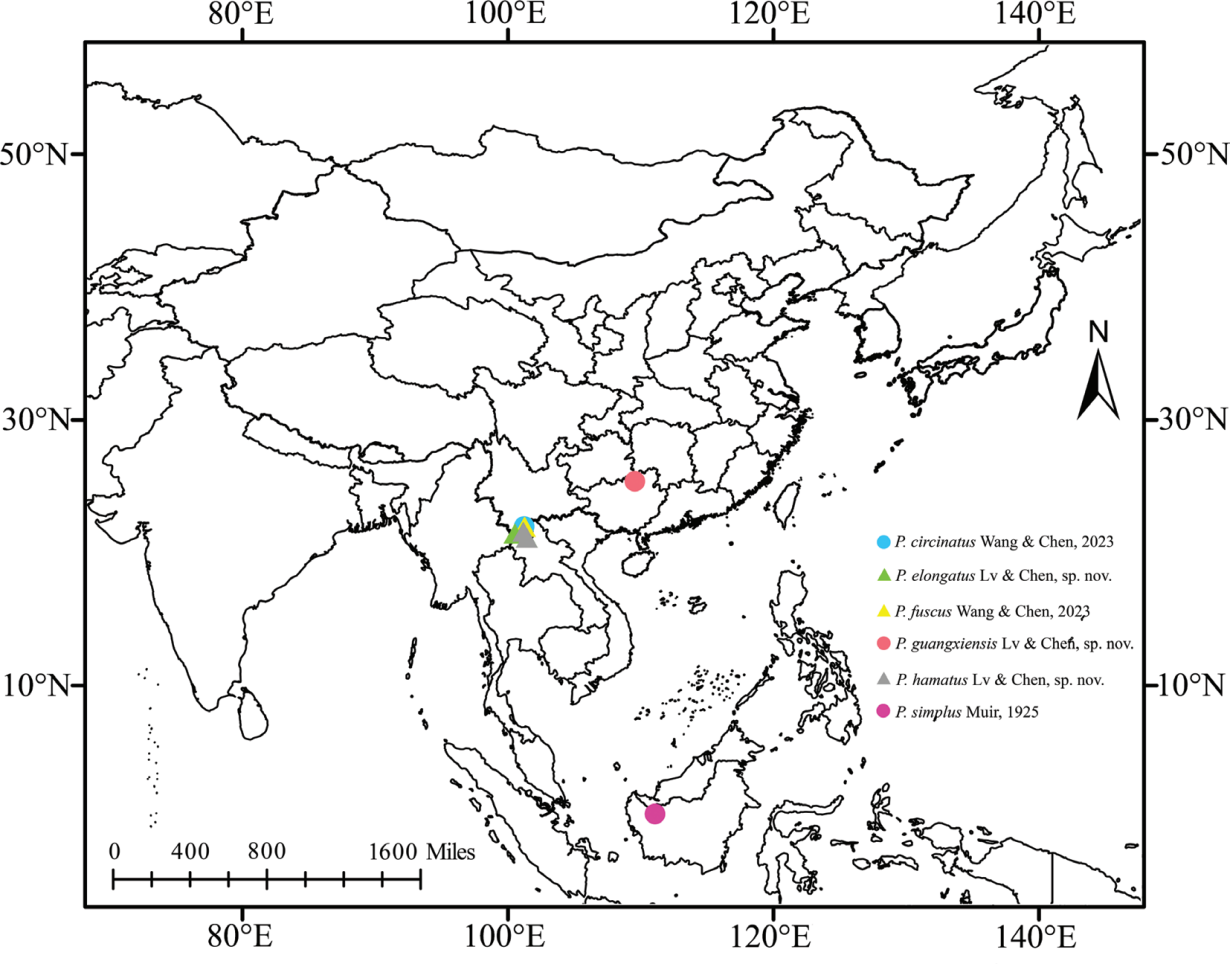
Geographic distributions of species of *Parandes* Muir, 1925.

China, Indonesia (Fig. 1).

##### ﻿Checklist and distributions of species of *Parandes* Muir, 1925

*P.circinatus* Wang & Chen, 2023; China (Yunnan Province).

*P.elongatus* Lv & Chen, sp. nov.; China (Yunnan Province).

*P.fuscus* Wang & Chen, 2023; China (Yunnan Province).

*P.guangxiensis* Lv & Chen, sp. nov.; China (Guangxi Zhuang Autonomous Region).

*P.hamatus* Lv & Chen, sp. nov.; China (Yunnan Province).

*P.simplus* Muir, 1925; Indonesia (West Borneo State).

### ﻿Key to species of *Parandes* Muir, 1925

Modified from Wang et al. 2023b.

**Table:** 

1	Forewing almost without markings; hind tibiae without lateral spines (Wang et al. 2023b: fig. 5A–C, G)	***P.simplus* Muir, 1925**
–	Forewing with markings; hind tibiae with several very small lateral spines	**2**
2	Forewing with a few small stripes at basal 2/3; apex dark brown, with a few pale spots (Wang et al. 2023b: fig. 3B, E)	***P.fuscus* Wang & Chen, 2023**
–	Forewing with several big variform markings at basal 2/3; apex light brown, with a few pale spots and small dark brown spots	**3**
3	Apex of gonostyli bent into a rectangular shape, inner margins with angular process in ventral view	**4**
–	Apex of gonostyli not bent into a rectangular shape, inner margins without angular process in ventral view	**5**
4	General color (Fig. 3A, B) light yellowish brown with green; inner margins of gonostyli (Fig. 3G) in ventral view sunken at base form a small process, middle part protrude slightly; long spinose process of periandrium (Fig. 3J–M) non-circular at apical part	***P.guangxiensis* Lv & Chen, sp. nov.**
–	General color (Fig. 5A, B) yellowish brown; inner margins of gonostyli (Fig. 5G) in ventral view with an angular process at middle part; long spinose process of periandrium (Fig. 5J–M) circular at apical part	***P.hamatus* Lv & Chen, sp. nov.**
5	Anal style (Fig. 2H) extending beyond anal segment; gonostyli (Fig. 2G) in ventral view fist-shaped at apex; periandrium (Fig. 2J–M) with a long stripy process at base to 2/3	***P.elongatus* Lv & Chen, sp. nov.**
–	Anal style not extending beyond anal segment; gonostyli in ventral view not fist-shaped at apex; periandrium without a long stripy process at base to 2/3 (Wang et al. 2023b: fig. 3G, H, J–M)	***P.circinatus* Wang & Chen, 2023**

### ﻿ Parandes elongatus

Taxon classificationAnimaliaHemipteraCixiidae

Lv & Chen sp. nov.

2B502B90-5ACF-5A90-88A8-201AD93ACF74

https://zoobank.org/3EE0A1C7-29D9-40BB-A916-74DF79D85021

Fig. 2

#### Type material.

***Holotype***: China • ♂: Yunnan Province, Mengla County, Mohan Town; 21°11'N, 101°41'E; sweeping, 11 August 2023; Sha-Sha Lv leg.; IEGU. ***Paratypes***: China • 1♂; Yunnan Province, Mengla County, Mohan Town; 21°11'N, 101°41'E; sweeping, 11 August 2023; Yong-Jin Sui leg.; IEGU. • 1♂; Yunnan Province, Mengla County, Menglun Town, Bakaxiaozhai Village; 21°58'N, 101°13'E; sweeping, 8 August 2023; Sha-Sha Lv leg.; IEGU. • 1♂; Yunnan Province, Jinghong City, Menglong Town, Guohe Village; 21°45'N, 100°51'E; sweeping, 13 August 2023; Feng-E Li leg.; IEGU.

#### Diagnosis.

**Figure 2.**
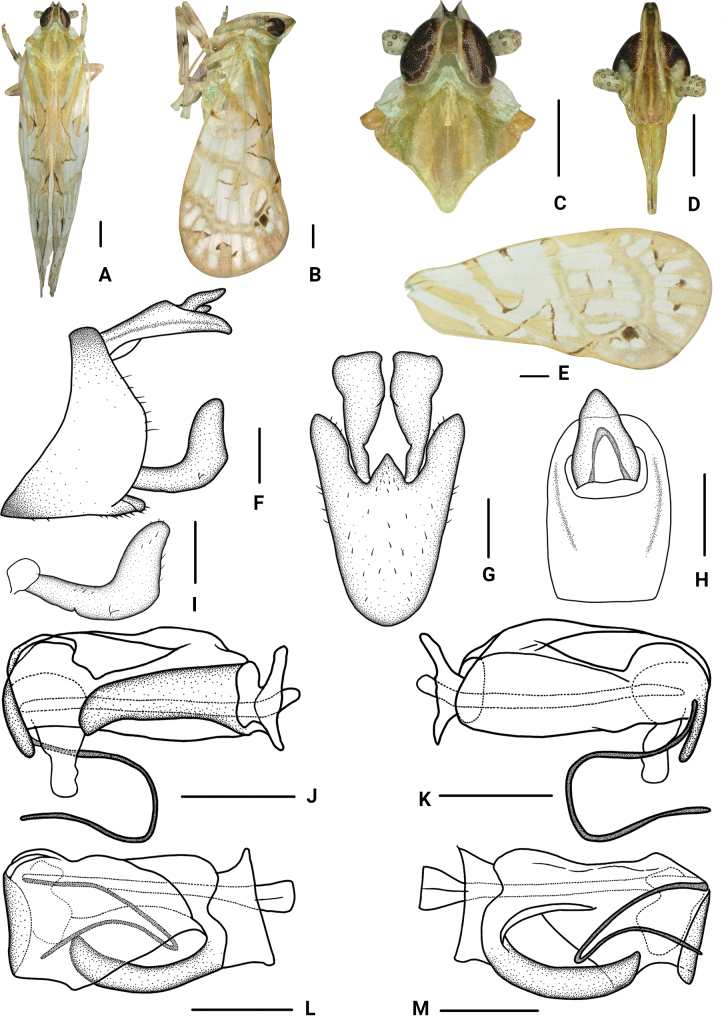
*Parandeselongatus* Lv & Chen, sp. nov., male. A. Habitus, dorsal view; B. Habitus, lateral view; C. Head and thorax, dorsal view; D. Frons, ventral view; E. Forewing; F. Genitalia, lateral view; G. Pygofer and gonostyli, ventral view; H. Anal segment, dorsal view; I. Gonostyli, lateral view; J. Aedeagus, right side; K. Aedeagus, left side; L. Aedeagus, dorsal view; M. Aedeagus, ventral view. Scale bars: 0.5 mm (A–E); 0.2 mm (F–M).

The salient features of the new species include: anal style (Fig. 2H) extending beyond anal segment; gonostyli in ventral view (Fig. 2G) fist-shaped at apex, basal 1/3 and 2/3 with emarginations; periandrium (Fig. 2J–M) in dorsal margin with a lamellar process, extending to apex to form a thin process, directed ventrad, base to 2/3 with a long stripy process.

#### Description.

***Measurements*.** Total length: male 6.1–6.4 mm (*N* = 4).

***Coloration*.** General color greyish white (Fig. 2A, B). Vertex yellowish with green. Eyes brown. Ocelli faint red. Frons yellowish brown, median carina dark yellow, lateral carinae pale yellowish green, lateral side of head with a triangular yellowish-green spot anterior to the eyes. Clypeus yellowish brown with green, carinae brown. Antennae grayish green. Pronotum with median part dark grayish green, lateral sides white with a little green. Mesonotum yellowish to brown. Tegula yellowish brown. Forewings semi-translucent, grayish white, with many variform pale yellowish green and dark brown stripes and markings, stigma light yellowish brown, veins and tubercles green to yellowish brown, as shown in Fig. 2E.

***Head and thorax*.** Vertex (Fig. 2A, C) 1.38 times as long as wide, width at apex narrower than at base (1:2.22), anterior margin nearly straight, posterior margin U-shaped recessed, lateral carina developed, median carina absent. Frons (Fig. 2D) longer in middle line than wide at widest portion (about 3.65:1), widest at nearly apex, lateral carina developed, apex of median carina raised. Clypeus (Fig. 2D) with distinct median carina. Pronotum (Fig. 2A, C) shorter than vertex in midline (1:1.57), posterior margin recessed. Mesonotum (Fig. 2A, C) longer than 2.31 times pronotum and vertex combined. Forewings (Fig. 2E) 2.19 times as long as wide, with twelve apical cells and seven subapical cells, RP 3 branches, MP with 5 terminals: MP_11_, MP_12_, MP_2_, MP_3_, and MP_4_, fork MP_1_+MP_2_ basad of fork MP_3_+MP_4_. Hind tibia with five lateral spines.

***Male genitalia*.** Pygofer (Fig. 2F, G) ventral margin distinctly longer than dorsal margin in lateral view, posterior margin convex at middle, lateral lobes arcuate and extended caudally; in ventral view symmetrical, medioventral process long, equilateral triangular. Anal segment (Fig. 2F, H) in lateral view flat tubular, dorsal margin straight, ventral margin curved slightly; in dorsal view, 1.48 times as long as wide; anal style big, triangular, extending beyond anal segment. Gonostyli (Fig. 2G, I) in lateral view L-shaped, curved dorsally near the middle, ventral margin with a notch at basal 1/3; in ventral view lateral margins curved, widens towards the end, fist-shaped at apex, basal 1/3 and 2/3 with emarginations. Aedeagus (Fig. 2J–M) with a spinose process. Periandrium in dorsal margin with a lamellar process, extending to apex, forming a thin process, directed ventrad; apical part with a slender spinous process, curved ventrally at base, then strong bending, directed caudad; base to 2/3 with a long stripy process. Endosoma slightly sclerotized, without process.

#### Distribution.

China (Yunnan Province) (Fig. 1).

#### Etymology.

The species name is derived from the Latin word “*elongatus*”, referring to periandrium with a long stripy process at base to 2/3.

#### Remarks.

This species is similar to *P.circinatus* Wang & Chen, 2023, but differs from the latter in: (1) anal style extending beyond anal segment (anal style not extending beyond anal segment in *P.circinatus*); (2) gonostyli in ventral view fist-shaped at apex (gonostyli in ventral view not fist-shaped at apex in *P.circinatus*); and (3) periandrium with a long stripy process at base to 2/3 (periandrium without a long stripy process at base to 2/3 in *P.circinatus*).

### ﻿ Parandes guangxiensis

Taxon classificationAnimaliaHemipteraCixiidae

Lv & Chen sp. nov.

178AF932-DFEF-5512-A161-BAF7BFFCFA5B

https://zoobank.org/3B8DCBC8-07D9-4A6B-9261-4316E006AB54

Figs 3 , 4

#### Diagnosis.

**Figure 3.**
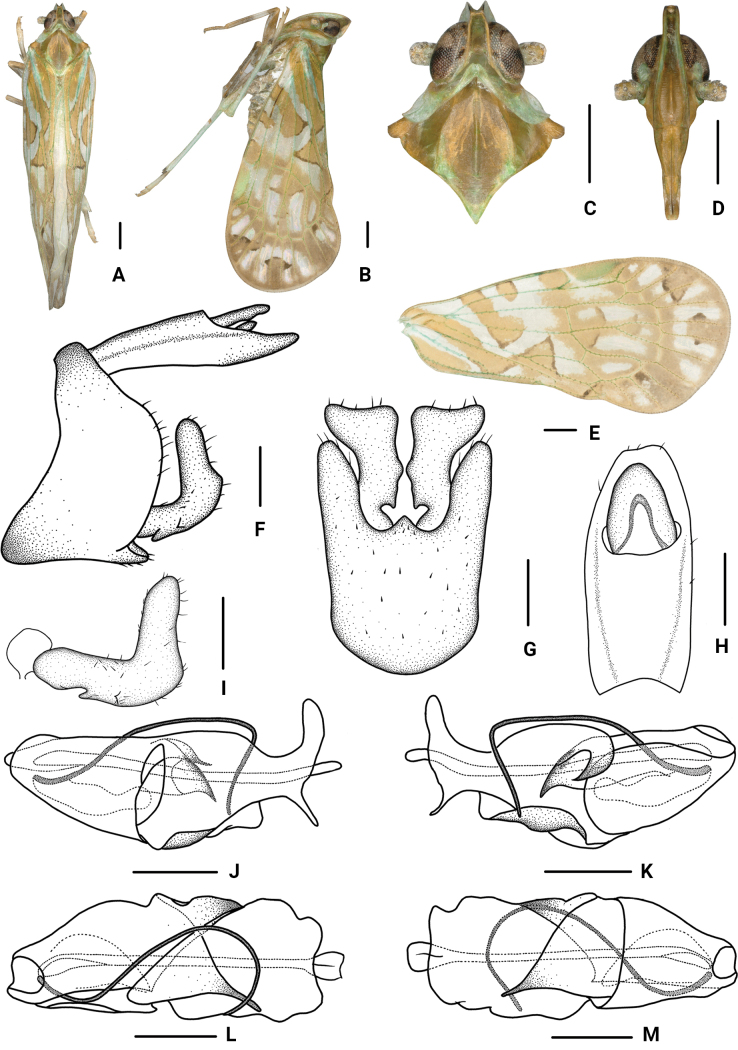
*Parandesguangxiensis* Lv & Chen, sp. nov., male. A. Habitus, dorsal view; B. Habitus, lateral view; C. Head and thorax, dorsal view; D. Frons, ventral view; E. Forewing; F. Genitalia, lateral view; G. Pygofer and gonostyli, ventral view; H. Anal segment, dorsal view; I. Gonostyli, lateral view; J. Aedeagus, right side; K. Aedeagus, left side; L. Aedeagus, dorsal view; M. Aedeagus, ventral view. Scale bars: 0.5 mm (A–E); 0.2 mm (F–M).

The salient features of the new species include: gonostyli in ventral view (Fig. 3G) bent into a rectangular shape at apex, inner margins sunken at base forming a small process, middle part protrudes slightly; periandrium (Fig. 3J–M) in ventral margin with a wide lamellar process at apical half, dorsal margin with a long strip-shaped process, tapering to apex, with a long hook-shaped process near the middle.

#### Type material.

***Holotype***: China • ♂; Guangxi Province, Huaping National Nature Reserve; 25°38'N, 109°55'E; sweeping, 9 August 2023; Yong-Jin Sui and Feng-E Li leg.; IEGU. ***Paratypes***: China • 15♂♂, 8♀♀; same collection data as for holotype; IEGU.

#### Description.

***Measurements*.** Total length: male 6.3–6.6 mm (*N* = 16), female 7.0–7.6 mm (*N* = 8).

***Coloration*.** General color yellowish brown with green (Fig. 3A, B). Vertex foul-brown with green, lateral carinae dark yellow. Eyes gray-brown to brown. Ocelli gray. Frons dark brown at base, rest yellowish brown, lateral carinae yellowish green to dark green, lateral side of head with a triangular turquoise spot anterior to the eyes. Clypeus yellowish brown. Antennae yellowish brown with green at base. Pronotum greyish white with turquoise, brown behind eyes. Mesonotum yellowish brown to brown with green. Tegula yellowish brown. Forewings semi-translucent, grayish white, with many variform yellowish brown to brown and dark brown stripes and markings, stigma yellowish green, veins and tubercles turquoise to green to light yellowish brown, as shown in Fig. 3E.

***Head and thorax*.** Vertex (Fig. 3A, C) 1.63 times as long as wide, width at apex narrower than at base (1:1.73), anterior margin nearly straight, posterior margin U-shaped recessed, lateral carina developed, median carina absent. Frons (Fig. 3D) longer in middle line than wide at widest portion (about 3.65:1), widest at nearly apex, lateral carina developed, apex of median carina raised. Clypeus (Fig. 3D) with median carina. Pronotum (Fig. 3A, C) shorter than vertex in midline (1:1.57), posterior margin recessed. Mesonotum (Fig. 3A, C) longer than 2.36 times pronotum and vertex combined. Forewings (Fig. 3E) 2.19 times as long as wide, with twelve apical cells and seven subapical cells, RP 3 branches, MP with 5 terminals: MP_11_, MP_12_, MP_2_, MP_3_, and MP_4_, fork MP_1_+MP_2_ basad of fork MP_3_+MP_4_. Hind tibia with six lateral spines.

***Male genitalia*.** Pygofer (Fig. 3F, G) ventral margin distinctly longer than dorsal margin in lateral view, posterior margin convex at middle, lateral lobes arcuate and extended caudally; in ventral view symmetrical, medioventral process short, triangular. Anal segment (Fig. 3F, H) in lateral view flat tubular, dorsal and ventral margins nearly straight; in dorsal view, 2.32 times as long as wide; anal style big, elliptic, not extending beyond anal segment. Gonostyli (Fig. 3I, G) in lateral view L-shaped, curved dorsally near the middle, ventral margin sunken at basal 1/3, with a small process; in ventral view lateral margins curved, bent into a rectangular-shape at apex, directed reversely, inner margins sunken at base form a small process, middle part protrudes slightly. Aedeagus (Fig. 3J–M) with a spinose process. Periandrium in ventral margin with a wide lamellar process at apical half, nearly basal part frizzy; dorsal margin with a long strip-shaped process, tapering to apex, with a long hook shaped near the middle, directed ventrocephalad; apical part with a slender spinous process, curved ventrally at base, slowly sloping to straight, then bends almost 90 degrees to the abdomen, directed ventrad. Endosoma slightly sclerotized, without process.

**Figure 4.**
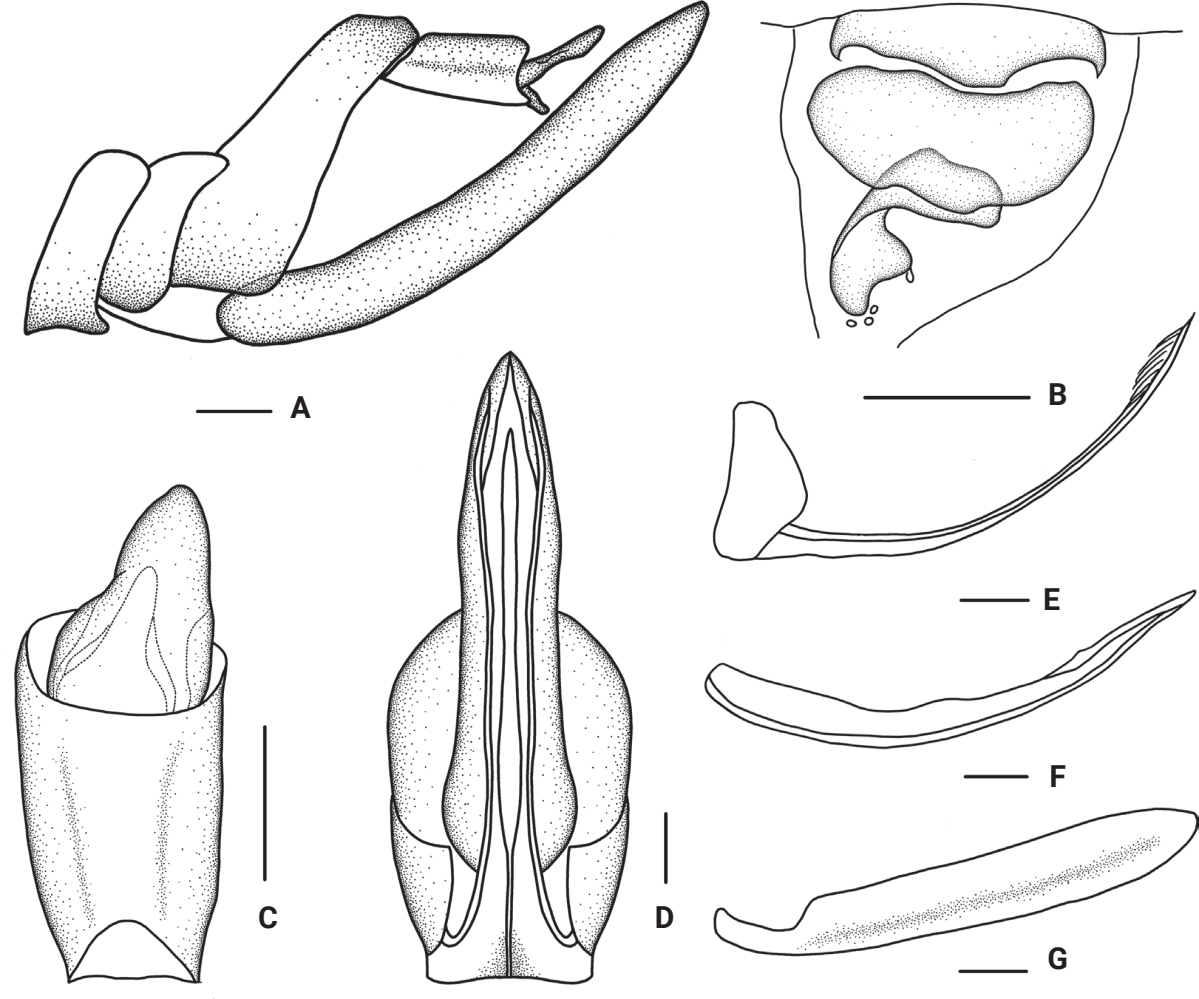
*Parandesguangxiensis* Lv & Chen, sp. nov., female. A. Genitalia, lateral view; B. Posterior vagina, ventral view; C. Anal segment, dorsal view; D. Genitalia, ventral view; E. Gonapophysis VIII and gonocoxa VIII, lateral view; F. Gonapophysis IX, lateral view; G. Gonoplac, inner lateral view. Scale bars: 0.2 mm (A–G).

***Female genitalia*.** Tergite IX (Fig. 4A, D) moderately sclerotized, without wax plate. Anal segment (Fig. 4C) rectangular, 1.80 times as long as wide in dorsal view, anal style angular. Gonapophysis VIII (Fig. 4E) elongate, curved upwards. Gonapophysis IX (Fig. 4F) with two middle teeth, denticulation unsharp. Gonoplac (Fig. 4G) rod-like, 6.65 times as long as wide in lateral view. Posterior vagina pattern as shown in Figure 4B.

#### Distribution.

China (Guangxi Zhuang Autonomous Region) (Fig. 1).

#### Etymology.

The new species is named after its the Guangxi Zhuang Autonomous Region in which it was collected.

#### Remarks.

This species is similar to *P.circinatus* Wang & Chen, 2023, but differs from the latter in: (1) gonostyli in ventral view bent into a rectangular shape at apex (gonostyli in ventral view not bent into a rectangular shape at apex in *P.circinatus*); (2) long spinose process of periandrium non-circular at apical part (long spinose process of periandrium circular at apical part in *P.circinatus*); and (3) periandrium with a long hook-shaped process near the middle (periandrium without a long hook-shaped process near the middle in *P.circinatus*).

### ﻿ Parandes hamatus

Taxon classificationAnimaliaHemipteraCixiidae

Lv & Chen sp. nov.

F91629BF-8413-518A-9FF6-B511FC6B69BF

https://zoobank.org/DEFE27BC-E931-41C4-8847-3BDB796B7D08

Figs 5 , 6

#### Type material.

***Holotype***: China • ♂; Yunnan Province, Mengla County, Menglun Town, Mangang Village; 21°58'N, 101°15'E; sweeping, 9 August 2023; Sha-Sha Lv leg.; IEGU. ***Paratypes***: China • 1♂1♀; Yunnan Province, Mengla County, Menglun Town, Mangang Village; 21°58'N, 101°15'E; sweeping, 9 August 2023; Feng-E Li and Sha-Sha Lv leg.; IEGU. • 2♂♂; Yunnan Province, Mengla County, Mohan Town; 21°12'N, 101°42'E; sweeping, 11 August 2023; Sha-Sha Lv leg.; IEGU.

#### Diagnosis.

**Figure 5.**
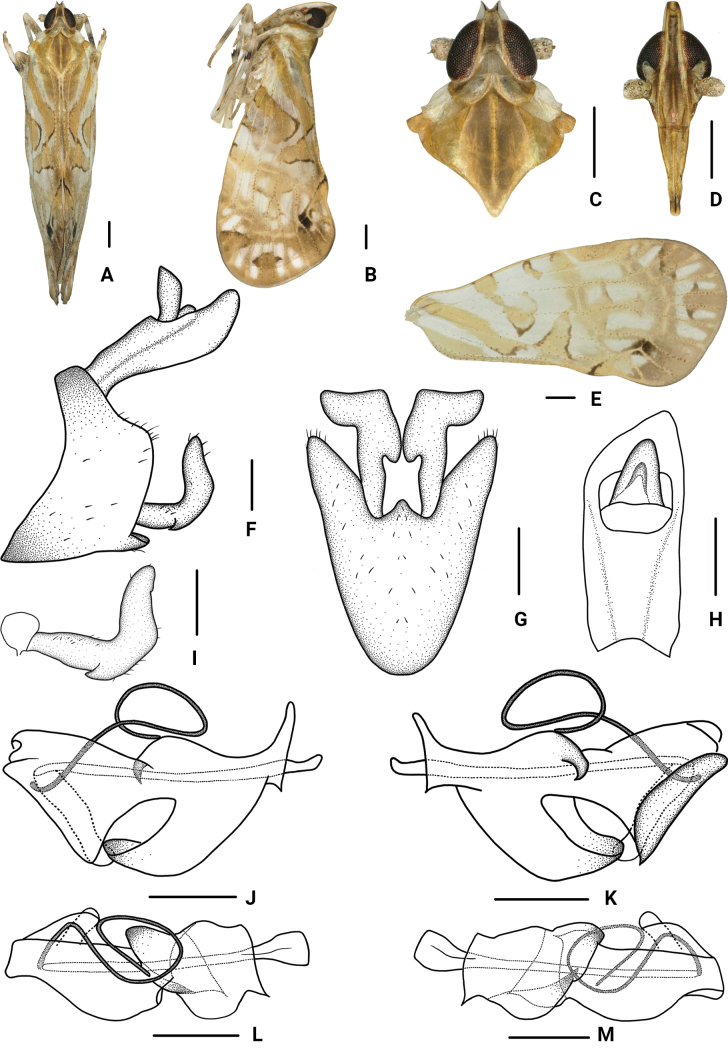
*Parandeshamatus* Lv & Chen, sp. nov., male. A. Habitus, dorsal view; B. Habitus, lateral view; C. Head and thorax, dorsal view; D. Frons, ventral view; E. Forewing; F. Genitalia, lateral view; G. Pygofer and gonostyli, ventral view; H. Anal segment, dorsal view; I. Gonostyli, lateral view; J. Aedeagus, right side; K. Aedeagus, left side; L. Aedeagus, dorsal view; M. Aedeagus, ventral view. Scale bars: 0.5 mm (A–E); 0.2 mm (F–M).

The salient features of the new species include: gonostyli in ventral view (Fig. 5G) bent into a rectangular shape at apex, with an angular process near the middle; dorsal margin of periandrium (Fig. 5J–M) with a hook-like process near the middle, ventral margin with a wide subquadrilateral process nearly basal to the middle part; slender spinose process of periandrium circular at apical part.

#### Description.

***Measurements*.** Total length: male 6.4–6.7 mm (*N* = 4), female 7.5 mm (*N* = 1).

***Coloration*.** General color brown (Fig. 5A, B). Vertex dark brown, lateral carinae yellowish brown. Eyes red-black. Ocelli faint red. Frons yellowish brown to dark brown, lateral carinae light yellowish brown at basal half, rest brown, lateral side of head with a grey-brown spot anterior to the eyes. Clypeus yellowish brown. Antennae gray-brown. Pronotum greyish white, yellowish brown behind eyes. Mesonotum brown at middle part, yellowish to yellowish brown at lateral sides. Tegula yellowish brown. Forewings semi-translucent, grayish white, with many variform yellowish brown to brown and dark brown stripes and markings, stigma yellowish brown, veins and tubercles greyish white to yellowish brown, as shown in Figure 5E.

***Head and thorax*.** Vertex (Fig. 5A, C) 1.51 times as long as wide, width at apex narrower than at base (1:2.22), anterior margin nearly straight, posterior margin U-shaped, recessed, lateral carina developed, median carina absent. Frons (Fig. 5D) longer in middle line than wide at widest portion (about 3.65:1), widest at nearly apex, lateral carina developed, apex of median carina raised. Clypeus (Fig. 5D) with distinct median carina. Pronotum (Fig. 5A, C) shorter than vertex in midline (1:1.57), posterior margin recessed. Mesonotum (Fig. 5A, C) longer than 1.76 times pronotum and vertex combined. Forewings (Fig. 5E) 2.19 times as long as wide, with twelve apical cells and seven subapical cells, RP 3 branches, MP with 5 terminals: MP_11_, MP_12_, MP_2_, MP_3_, and MP_4_, fork MP_1_+MP_2_ basad of fork MP_3_+MP_4_. Hind tibia with six lateral spines.

***Male genitalia*.** Pygofer (Fig. 5F, G) ventral margin distinctly longer than dorsal margin in lateral view, posterior margin with middle part distinctly convex, lateral lobes arcuate and extended caudally; in ventral view symmetrical, medioventral process short, angular. Anal segment (Fig. 5F, H) in lateral view flat tubular, dorsal margin almost straight, ventral margin curved slightly; in dorsal view apical part angular, 2.42 times as long as wide; anal style tonguelike, big, not extending beyond anal segment. Gonostyli (Fig. 5I, G) in lateral view curved dorsally, L-shaped, ventral margin with a dentoid process, directed cephalad; in ventral view bent into a rectangular shape at apex, directed reversely, middle part of inner margins broad suddenly, with an angular process. Aedeagus (Fig. 5J–M) with a spinose process. Periandrium in ventral margin with a suboblong lamellar process at apical part, nearly basal to the middle part with a wide subquadrilateral process; dorsal margin with a hook-like process near the middle, tapering to apex, directed ventrocephalad; apical part with a slender process, curved ventrally at base, slowly sloping, then curved in a circle from middle part to proximal apex, directed cephalad. Endosoma slightly sclerotized, without process.

**Figure 6.**
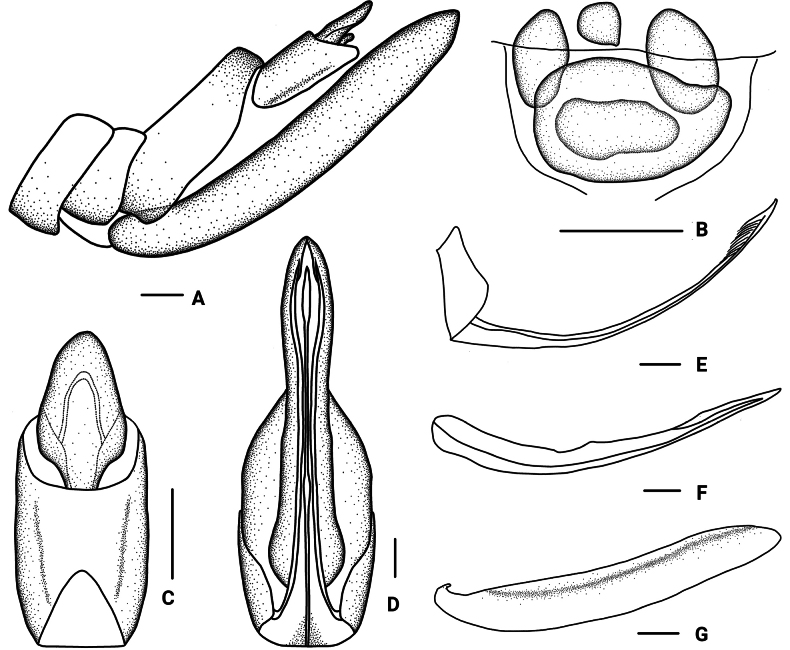
*Parandeshamatus* Lv & Chen, sp. nov., female. A. Genitalia, lateral view; B. Posterior vagina, ventral view; C. Anal segment, dorsal view; D. Genitalia, ventral view; E. Gonapophysis VIII and gonocoxa VIII, lateral view; F. Gonapophysis IX, lateral view; G. Gonoplac, inner lateral view. Scale bars: 0.2 mm (A–G).

***Female genitalia*.** Tergite IX (Fig. 6A, D) moderately sclerotized, without wax plate. Anal segment (Fig. 6C) rectangular, 1.82 times as long as wide in dorsal view, anal style egg-shaped. Gonapophysis VIII (Fig. 6E) elongate, curved upwards. Gonapophysis IX (Fig. 6F) with two middle teeth, denticulation unsharp. Gonoplac (Fig. 6G) rod-like, 6.32 times as long as wide in lateral view. Posterior vagina pattern as shown in Figure 6B.

#### Distribution.

China (Yunnan Province) (Fig. 1).

#### Etymology.

The species name is derived from the Latin word “*hamatus*”, referring to the dorsal margin of the periandrium with a hook-like process near the middle.

#### Remarks.

This species is similar to *P.fuscus* Wang & Chen, 2023, but differs from the latter in: (1) forewing with several large variform markings at basal 2/3, apex light brown, with a few pale spots and small dark brown markings (forewing with a few small stripes at basal 2/3, apex dark brown, with a few pale spots in *P.fuscus*); (2) gonostyli bent into a rectangular shape at apex (gonostyli not bent into rectangular shape at apex in *P.fuscus*); and (3) dorsal margin of periandrium with a hook-like process near the middle (dorsal margin of periandrium without a hook-like process near the middle in *P.fuscus*).

## ﻿Discussion

Andini represents a small group within Cixiidae, comprising three genera: *Andes* Stål, 1866, *Parandes* Muir, 1925, and *Andixius* Emeljanov & Hayashi, 2007. These genera are closely similar in appearance. The genus *Andixius* can be distinguished by the specific characteristics of the forewings without trifid branching of ScP+R and MP near basal cell, ScP+R (ScP+RA and RP) forming a short common stalk (Wang et al. 2020). However, the genera *Andes* and *Parandes* are differentiated by the distinctive features of the fore coxa produced and rounded on the outer edge of the apical half, which are challenging to discern. Wang et al. (2023b) have also discussed the diagnostic traits that distinguish this genus from others. In this study, we have described three new species, which, along with the existing species, share common features that distinguish them from the other two genera: head in profile with junction of vertex and frons slightly angular and slightly produced; fore coxa produced and rounded on the outer edge of apical half; apex of the periandrium with a long spinose process; and endosoma simple, without a process.

The Andini in China comprises 26 species, representing approximately 19.26% of the global total of 135 species, and is distributed in the Sino-Japanese–Oriental Region (NC, QT, SWC, CC, SC, TW). The tribe serves as a landmark for the Palearctic/Sino-Japanese north boundary (Luo et al. 2022). The genus *Parandes* is known to contain only three species. However, based on the findings of this research, the total count of species within the genus has been updated to six. At present, all species are distributed in the Oriental region (Fig. 1). Except for *P.simplus* Muir, 1925, which has been documented in Indonesia, the remaining five species are predominantly found in China. Within China, aside from *P.guangxiensis* sp. nov., which is specific to Guangxi, the other four species are primarily distributed in Yunnan. There is no disputing that Yunnan’s unique geographical position and climate contribute to its rich biodiversity. Nevertheless, the discovery of new species in Guangxi has expanded the known distribution beyond Yunnan, highlighting the necessity for further extensive species surveys.

Cixiids are sap-sucking insects that feed on a wide range of plants, encompassing over 51 orders, including Alismatales, Apiales, Aquifoliales, Arecales, Asparagales, Asterales, Boraginales, Brassicales, Canellales, Caryophyllales, Celastrales, Chloranthales, Cornales, Crossosomatales, Cucurbitales, Cupressales, Cyatheales, Cycadales, Dilleniales, Ericales, Fabales, Fagales, Gentianales, Geraniales, Gleicheniales, Hamamelidales, Lamiales, Laurales, Liliales, Linales, Malvales, Malpighiales, Myrtales, Oxalidales, Pandanales, Plumbaginales, Pinales, Piperales, Podocarpales, Poales, Polypodiales, Proteales, Ranunculales, Rosales, Sapindales, Saxifragales, Scrophulariales, Solanales, Theales, Vitales, and Zingiberales (Bertin et al. 2010; Löcker and Holzinger 2019; Luo et al. 2019; Chen et al. 2024; Chen and Yang 2024; Bourgoin 2025). The host plants of Andini have not yet been clearly documented. Wang et al. (2022) suggested that the potential hosts for the genus *Andes* might be dark and damp mosses and ferns. During our field trips, *Parandes* were mostly collected from small macrophanerophytes, which may serve as their hosts.

## Supplementary Material

XML Treatment for Parandes

XML Treatment for Parandes elongatus

XML Treatment for Parandes guangxiensis

XML Treatment for Parandes hamatus

## ﻿Additional information

### Use of AI

No use of AI was reported.

### Author ORCIDs

Sha-Sha Lv https://orcid.org/0000-0001-5353-5082

Nian Gong https://orcid.org/0000-0002-8878-5337

Xiao-Ya Wang https://orcid.org/0000-0002-0978-2470

Lin Yang https://orcid.org/0000-0002-7841-5156

Yu-Bo Zhang https://orcid.org/0000-0002-6118-6190

Xiang-Sheng Chen https://orcid.org/0000-0001-9801-0343

### Data availability

All of the data that support the findings of this study are available in the main text.
